# Considering the *APOE* locus in Alzheimer’s disease polygenic scores in the Health and Retirement Study: a longitudinal panel study

**DOI:** 10.1186/s12920-020-00815-9

**Published:** 2020-11-03

**Authors:** Erin B. Ware, Jessica D. Faul, Colter M. Mitchell, Kelly M. Bakulski

**Affiliations:** 1 Survey Research Center, Institute for Social Research, University of Michigan, 426 Thompson St., Rm. 3320 ISR-Thompson, Ann Arbor, MI 48104 USA; 2 Department of Epidemiology, School of Public Health, University of Michigan, Ann Arbor, MI 48109 USA

**Keywords:** Polygenic score, Alzheimer’s disease, Dementia, Apolipoprotein E, P-value, Thresholding

## Abstract

**Background:**

Polygenic scores are a strategy to aggregate the small, additive effects of single nucleotide polymorphisms across the genome. With phenotypes like Alzheimer’s disease, which have a strong and well-established genomic locus (*APOE*), the cumulative effect of genetic variants outside of this area has not been well established in a population-representative sample.

**Methods:**

Here we examine the association between polygenic scores for Alzheimer’s disease both with and without the *APOE* region (chr19: 45,384,477 to 45,432,606, build 37/hg 19) at different *P* value thresholds and dementia. We also investigate the addition of *APOE*-ε4 carrier status and its effect on the polygenic score—dementia association in the Health and Retirement Study using generalized linear models accounting for repeated measures by individual and use a binomial distribution, logit link, and unstructured correlation structure.

**Results:**

In a large sample of European ancestry participants of the Health and Retirement Study (n = 9872) with an average of 5.2 (standard deviation 1.8) visit spaced two years apart, we found that including the *APOE* region through weighted variants in a polygenic score was insufficient to capture the large amount of risk attributed to this region. We also found that a polygenic score with a *P* value threshold of 0.01 had the strongest association with the odds of dementia in this sample (odds ratio = 1.10 95%CI 1.0 to 1.2).

**Conclusion:**

We recommend removing the *APOE* region from polygenic score calculation and treating the *APOE* locus as an independent covariate when modeling dementia. We also recommend using a moderately conservative *P* value threshold (e.g. 0.01) when creating polygenic scores for Alzheimer’s disease on dementia. These recommendations may help elucidate relationships between polygenic scores and regions of strong significance for phenotypes similar to Alzheimer’s disease.

## Background

The most common form of dementia is Alzheimer’s disease (AD), represented in roughly 65% of dementia cases [1]. Alzheimer’s disease is thought to arise from a combination of both genetics, environment, and lifestyle factors [2]. The estimated heritability of late onset Alzheimer’s disease is around 74% [3]. While large-scale genome-wide association studies (GWAS) have identified several genetic loci associated with Alzheimer’s disease [4–10], being a carrier of the Apolipoprotein E (*APOE-ε4*) allele remains the strongest genetic predictor of late-onset Alzheimer’s disease [11]. One copy of *APOE-ε4* (inheriting a CC at these two locations from either parent) confers a threefold risk of Alzheimer’s disease while two copies (inheriting a CC at these two locations from *both* parents) a 15-fold increase in risk [12]. The effect of *APOE-ε4* is all the more difficult to capture in a single variant GWAS as *APOE-ε4* is a haplotype composed of two SNPs: rs7412 and rs429358—which will never be fully be captured in a traditional linear model GWAS framework. However, GWAS have identified many independent SNPs in and near the *APOE* gene locus. The *APOE* gene region contains many variants in high linkage disequilibrium within roughly 100 kilobases, including several additional high-risk sites in the translocase of outer mitochondrial membrane 40 (*TOMM40*) gene.

The largest Alzheimer’s disease GWAS meta-analysis to date (N = 94,437) is the from the International Genomics of Alzheimer’s Project (IGAP) [6]. This meta-analysis confirmed 20 previously identified Alzheimer’s disease risk loci [4] and identified five new genome-wide loci including (*IQCK*, *ACE*, *ADAM10*, *ADAMTS1*, and *WWOX*). The IGAP used a three-stage strategy where Stage 1 consisted of genotyped and imputed data on 9,456,058 common and 2,024,574 rare single nucleotide polymorphisms (SNPs) to meta-analyze GWAS from four cohorts (n_cases_ = 21,982; n_controls_ = 41,944). Stage 2 included replication with a custom I-select genotyping chip developed in Lambert et al. 2013 [4] and included 11,632 variants and 18,845 individuals with a meta-analysis of Stage 1 and Stage 2. Finally, Stage 3 replicated 44 variants and meta-analyzed Stages 1 and 2 and 3 for a total of 35,274 cases and 59,163 controls. The associations between millions of genetic loci and Alzheimer’s disease are documented in IGAP and available for testing in independent populations.

Many complex diseases may result from the consideration of small individual effects across the genome. Polygenic scores (PGS)—also known as polygenic risk scores/PRS, though they do not always model a risky phenotype (e.g. “risk” of high education, “risk” of increased height)—are generally derived from the sum of weighted variants across an individual [13, 14]. PGS incorporate genome-wide genetic variation into a single, quantitative measure that can be used in modeling as a tool to assess susceptibility. Though conceptually simple, many analytic decisions contribute to different qualities of PGSs including different coefficient of variation (R^2^), correlations between scores, and areas under the curve. An important consideration for diseases such as Alzheimer’s disease—which have a genetic locus like *APOE/TOMM40* conferring much of the genetic risk to the disease—is to determine how the remaining variants in the genome contribute to the disease. Removing a region with many risk variants and deciding which and how many variants to include in a PGS can offer substantively different conclusions. For instance, one study reported a PGS area under the curve of 0.57 for Alzheimer’s disease (parental proxy) using 21 SNPs and excluding the *APOE* region [7], while another study reported using more than 200,000 variants (including *APOE*) and a PGS area under the curve of 0.84 for Alzheimer’s disease [15]. While Alzheimer’s disease has a strong genetic locus, there is no benchmark across metrics of PGS construction for the rest of the genome’s polygenic contribution.

Population-based studies often assess dementia status, rather that AD, as a trade-off between feasibility for longitudinal, larger samples of more diverse participants versus more specific and intensive clinical assessments. An Alzheimer’s disease PGS may be informative for dementia more broadly, and Alzheimer’s disease PGS has not been assessed in population-based studies of dementia, other than by proxy in the UK Biobank cohort [5]. Further PGS construction metrics regarding the *APOE* region and additional SNPs have not been compared. The goals of this manuscript are three-fold. The first aim is to assess the utility of using Alzheimer’s disease PGSs in population-based analyses of dementia. The second aim is to evaluate the inclusion of the *APOE* region in these PGSs with and without a covariate modeling risk directly from the *APOE-ε4* allele. The third aim is to test SNP inclusion thresholds in PGS on dementia. We conduct this analysis using the Health and Retirement Study (HRS) in the European ancestries (intentionally plural, as there is no single “European” ancestry) participants.

## Methods

### Health and Retirement Study

The Health and Retirement Study (HRS) is a nationally representative panel study featuring a biennial survey of adults over age 50 and their spouses in the United States [16]. The HRS is sponsored by the National Institute on Aging (NIA U01AG009740) and is conducted by the University of Michigan. The HRS began in 1992 as a means to provide a national resource for data on changing health and economic circumstances associated with ageing at both the individual and population levels. These changes are focused on four broads topics: income and wealth; health, cognition, and use of healthcare services; work and retirement; and family connections [17].

The HRS pre-selected a random one-half of the sample to receive an enhanced face-to-face interview in 2006, which included physical performance tests, anthropometric measurements, blood and saliva samples, and a psychosocial self-administered questionnaire in addition to the HRS core interview. The remaining one-half sample received the same enhanced face-to-face protocol in 2008. The HRS randomly assigned the new 2010 cohort to receive an enhanced face-to-face interview in either 2010 or 2012. Those participants who were not interviewed or did not consent to saliva in 2006 were asked again in 2010.

Salivary DNA was collected using Oragene-250 saliva kits and protocol. DNA extracted from the saliva and was genotyped at the Center for Inherited Disease Research (CIDR) using the Illumina HumanOmni2.5 array (8v1 and 4v1). The Genetics Coordinating Center at the University of Washington, Seattle, WA performed Genotyping Quality Control. SNP annotation aligned to genome build 37/hg 19. The Genetics Coordinating Center calculated genetic principal components (PC) with HapMap controls [18, 19]. In addition to selecting independent SNPs with missing call rates < 5% and minor allele frequencies > 5%, the 2q21 (LCT), HLA, 8p23, and 17q21.31 regions were excluded from the initial pool [20]. The final European ancestries sample includes all self-reported non-Hispanic White persons that had PC loadings within ± one standard deviations of the mean for eigenvectors one and two in the PC analysis of all unrelated study subjects. The HRS re-calculated genetic PCs within the group of non-Hispanic Whites of European ancestries to further account for population stratification. These “ancestry-specific principal components” were used in subsequent analyses. Imputation was performed using IMPUTE2 on HRS data phased using SHAPEIT2. Data were imputed to the 1000 Genomes Project (1000GP) cosmopolitan reference panel phase 3 version 5 (initial release on May 2013, haplotypes released Oct 2014) and are available on the database of genotypes and phenotypes (dbGaP, https://www.ncbi.nlm.nih.gov/gap/,phs000428.v2.p2).

## APOE-ε4

The apolipoprotein E (*APOE*) gene codes for a protein that binds and transports low-density lipids and is responsible, in part, for removing cholesterol from the bloodstream [21, 22]. Variations in this gene affect cholesterol metabolism and may lead to increases in the risk for stroke, heart disease, and may alter the odds of having Alzheimer’s disease. Two variants (rs7412 and rs429358) define the APOE genotype resulting in three common isoforms of protein *apoE*: E2, E3, and E4 encoded by *ε2*, *ε3*, and *ε4*. Of note, the genotyped rs7412 and rs429358 variants failed genotyping quality control in the HRS pipeline and are therefore not included as individual variants in any PGS using genotyped data alone. Using the imputed rs7412 (IMPUTE2 INFO score = 0.99) and rs429358 (IMPUTE2 INFO score = 0.99) variants, we categorized HRS participants as *ε2/ε2*, *ε2/ε3*, *ε2/ε4*, *ε3/ε3*, *ε3/ε4*, and *ε4/ε4*. We analyze two indicator variables for presence of one *ε4* allele (1 = yes, 0 = no) or two *ε4* allele (1 = yes, 0 = no).

## Polygenic score for Alzheimer’s disease

We investigate using genome-wide raw genotyped variants and the two imputed *APOE* variants (rs7412 and rs429358) in the creation of our PGSs. We include SNPs in our PGS analysis at six AD-SNP association *P*-value thresholds (pT) from the Kunkle et al. [6] summary statistics: pT = (0.001, 0.01, 0.05, 0.1, 0.3, 1.0). For example, a PGS at pT = 0.01 includes only those variants for which the association p-value in the Kunkle et al. [6] GAP meta-analysis on Alzheimer’s disease was less than 0.01. We obtained summary statistics from National Institute on Aging Genetics of Alzheimer's Disease Data Storage Site https://www.niagads.org/datasets/ng00075. Importantly, our study sample was not included in the Kunkle study of clinical Alzheimer’s disease. Thus, the weights are independent of our study sample. We do not include any linkage disequilibrium filtering in our scores (i.e. no clumping or pruning algorithms). That is, we include any directly genotyped variants that pass quality control from the HRS and overlap with those variants from the Alzheimer’s disease GWAS summary statistics in our scores, unless otherwise noted. We did not perform clumping or pruning because we are using the set of tag-SNPs from the genotyping chip, which do not contain imputed variants and represent, in essence, an already filtered set of variants.

**Fig. 1:**
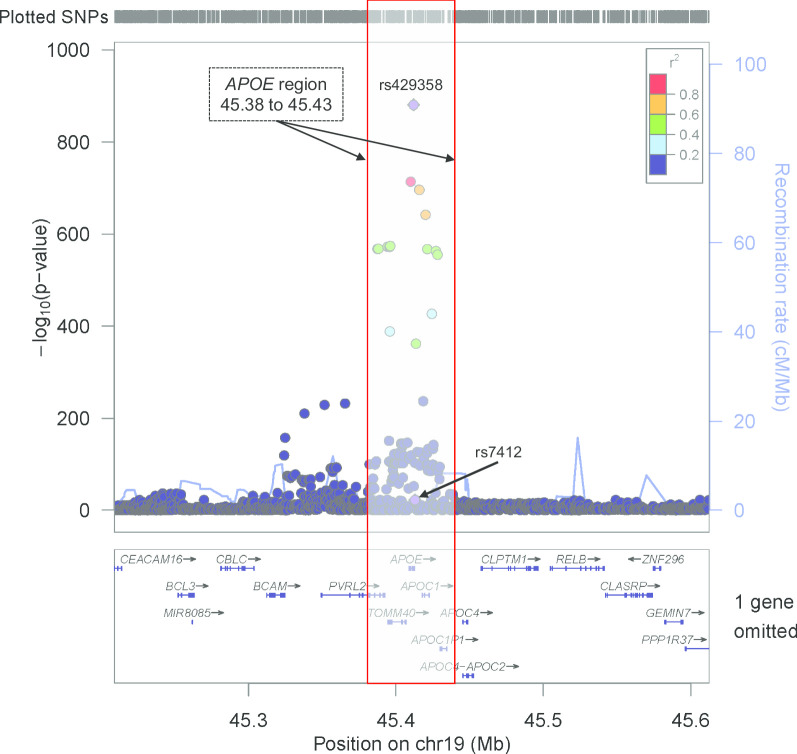
The *APOE/TOMM40* genomic locus on chromosome 19. The y-axis corresponds to − log_10_(p-values) of association with Alzheimer’s disease in Kunkle et al. [6]. Single nucleotide polymorphisms within the bracketed genomic region were removed from consideration in polygenic score development for scores designed to exclude the *APOE* region. The variants that make the *APOE* isoforms are highlighted in purple diamond (rs7412, rs429358)

For our scores with the *APOE* gene region removed, we removed all variants from the summary statistics on chromosome 19 near *APOE* (45,384,477 to 45,432,606, build 37/hg 19). This represents the start position of *TOMM40* (45,394,477) − 10 kilobases and the stop position of *APOC1* (45,422,606) + 10 kilobases. This region was removed in its entirety due to the dense linkage disequilibrium block in European ancestries overlapping these three genes (*TOMM40*, *APOE*, *APOC1*). We compare two PGSs: genotyped PGS including the *APOE* gene region, genotyped PGS with *APOE* region removed (Fig. 1), at six p-values thresholds from the Kunkle et al. [6] GWAS [(pT = (0.001, 0.01, 0.05, 0.1, 0.3, 1.0)].

## Cognition status

The HRS used a multidimensional measure of cognitive functioning, based on a telephone screening instrument: Telephone Interview for Cognitive Status [23]. Domains assessed using this measure include: memory, mental status, abstract reasoning, fluid reasoning, vocabulary, dementia, and numeracy. In 2009, Langa, Kabeto, and Weir developed an approach for defining dementia and cognitively impaired non-dementia (CIND) in the HRS. A team of dementia experts clinically validated this method using equipercentile equating in the HRS against the Aging, Demographics, and Memory Study (ADAMS). The ADAMS study is a sub-sample of the HRS who received a more extensive neurological battery [24, 25]. For self-respondents, the score consists of overall cognitive test performance while the proxy respondents’ scores are composed of proxy-rated memory, interviewer-perceived cognition, and impaired activities of daily living. The cut points for this method reflect the prevalence of dementia or cognitive impairment to the expected population prevalence from the ADAMS study. We used repeated measures of the classification of cognitive function data contributed for assessment years 2000−2014 from the HRS imputed cognition researcher contribution data set [26]. For self-respondents, a score from 0 to 6 is categorized as dementia, 7 to 11 is categorized as cognitive impaired not dementia, and 12 to 27 is categorized as normal cognition. For proxy respondents starting in 2000, a score of 6 or higher out of 11 is classified as having dementia, a score of 3 to 5 indicates cognitive impaired not dementia, while 0 to 2 indicates normal cognition [24]. In this analysis, we are only testing the odds of dementia versus normal cognition (1 = dementia, 0 = normal cognition).

## Covariates

Educational attainment (years of school), birth cohort [(AHEAD: Asset and Health Dynamics Among the Oldest Old (b. < 1924); CODA: Children of the Depression (b. 1924–1930); HRS: Health and Retirement Study—original cohort (b. 1931–1941); WB: War babies (b. 1942–1947); EBB: early baby boomers (b. 1948–1953); MBB: mid-baby boomers (b. 1954–1959)], and sex (0 = female, 1 = male) are measured at a participant’s initial HRS exam. Age (years) and a self-report of doctor diagnosed stroke (0 = none, 1 = stroke, possible stroke/TIA/mini-strokes, respondent disputes previous waves that indicate condition) are assessed at the same wave as the corresponding cognition visit.

## Statistical analysis

Due to the repeated measures in this analysis, we use generalized linear models and the GENMOD procedure in SAS 9.4. We specify repeated measures on the individual, a binomial distribution, and a logit link with an unstructured correlation structure. We include fixed effects covariates in every model: sex, years of education, and five ancestry-specific principal components. The time varying covariates chronological age, year, and stroke history at each interview wave are also included in every model. To evaluate the utility of the PGS in individuals with and without *APOE-ε4*, we also investigate an interaction effect between *APOE-ε4* status and PGS. We use an α of 0.05 as a threshold for significance.

## Results

**Fig. 2:**
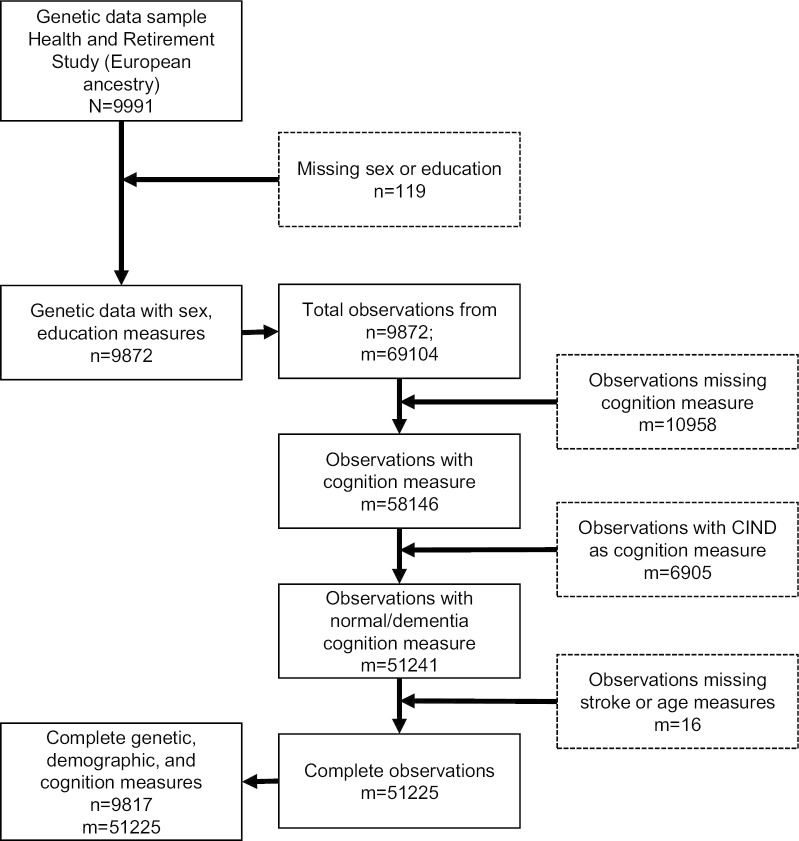
Sample inclusion flow chart for the Health and Retirement Study. HRS: Health and Retirement Study; n: number of individuals; m: number of observations; CIND: cognitively impaired, non-dementia

There are 9,872 individuals in the HRS non-Hispanic White, European ancestries analytic sample collected between 2006 and 2010. We removed observations with missing cognition (m = 10,958), observations where the cognitive status was classified as CIND (m = 6905), and observations with a missing stroke status (m = 16). This removed 55 individuals from the analysis. The final analysis included n = 9817 h respondents of European ancestries with a total of m = 51,225 cognitive observations (Fig. 2).

**Fig. 3:**
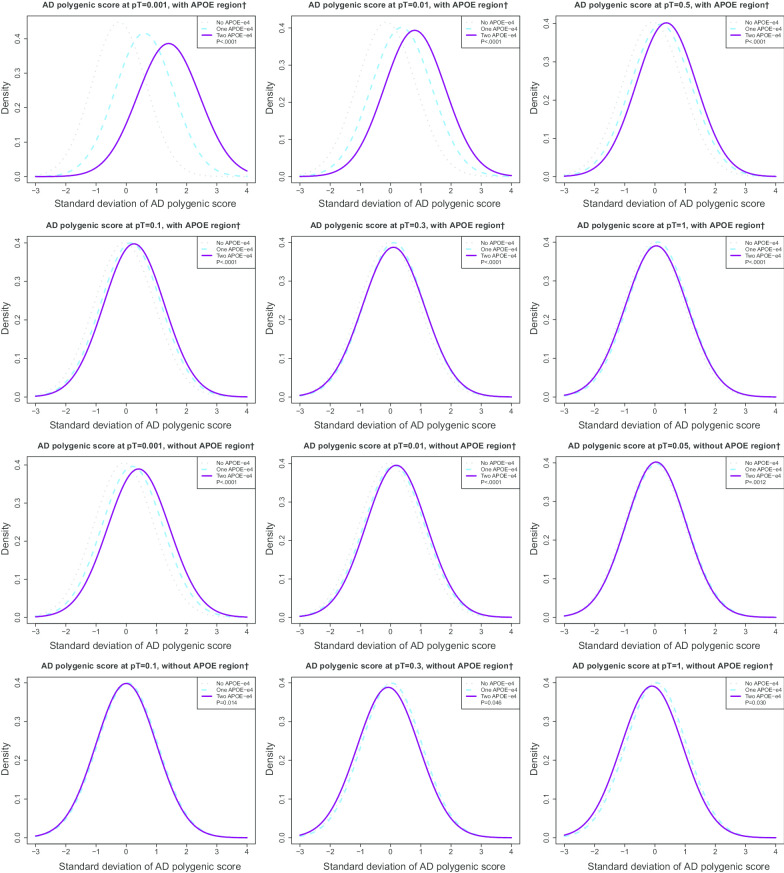
Distribution of Alzheimer’s disease polygenic score, by number of copies of *APOE-ε4* in the Health and Retirement Study, n = 9871. AD: Alzheimer’s disease; pT: p-value threshold for SNP-outcome association from the Alzheimer’s disease meta-analysis for inclusion into the polygenic score. ^†^*APOE* region defined as chromosome 19 (45,384,477 to 45,432,606, build 37/hg 19). This represents the start position of *TOMM40* (45,394,477) − 10 KB and the stop position of *APOC1* (45,422,606) + 10 KB

Our analytic sample was 57.8% female with an average age of 63.3 (SD = 10.3) at their first visit during the observation period. On average, participants had 13.2 (2.5) years of education. The average number of cognitive assessments per participant was 5.2 (1.8). A small portion of the sample had a history of stroke at their first visit during the observation period (n = 417, 4.3%), with a higher proportion of males reporting a history of stroke at their first visit (n = 201, 4.9%) than females (n = 216, 3.8%). In unadjusted analyses, the Alzheimer’s disease PGS at any pT was not significantly different between males and females and broadly centered at zero with a standard deviation of one. There was no difference in the distribution of number of copies of *APOE-ε4* by sex, where the overall proportion with one copy of *APOE-ε4* was 24.3% (n = 2382), and two copies of *APOE-ε4* was 2.1% (n = 210). Across all observations, accounting for repeated measures within individuals, age and dementia at first visit during the observation period were not significantly different by sex. The proportion of stroke; however, was significantly different by sex (*P* = 0.0004) with a higher proportion in males than in females (Table 1). *APOE-ε4* status is associated (all *Ps* < 0.05) with PGS (Fig. 3). Additional file 1: AF Table 1 contains a table of correlations between each Alzheimer’s disease PGS.

**Table 1:** Individual and observation-level descriptive statistics in the Health and Retirement Study, n = 9871, m = 51225

	Individuals			
	Male	Female	Overall	P^†^
	n = 4141	n = 5676	n = 9817	
Number of visits	5.0 (1.8)	5.4 (1.7)	5.2 (1.8)	< .0001
Dementia at first visit, n (%)	121 (2.9)	135 (2.4)	256 (2.6)	0.1
Age (yrs) first visit	63.7 (9.7)	63.0 (10.8)	63.3 (10.3)	< .001
Education (yrs)	13.4 (2.7)	13.1 (2.4)	13.2 (2.5)	< .0001
*HRS Cohort, n (%)*				< .0001
AHEAD	756 (13.3)	310 (7.5)	1066 (10.9)	
CODA	552 (9.7)	348 (8.4)	900 (9.2)	
HRS	2573 (45.3)	1928 (46.6)	4501 (45.9)	
WB	625 (11)	529 (12.8)	1154 (11.8)	
EBB	793 (14)	690 (16.7)	1483 (15.1)	
MBB	377 (6.6)	336 (8.1)	713 (7.3)	
Stroke at first visit, n (%)	201 (4.9)	216 (3.8)	417 (4.3)	0.01
*APOE-ε4, n (%)*				0.98
No copies	3051 (73.7)	4174 (73.5)	7225 (73.6)	
One copy	1001 (24.2)	1381 (24.3)	2382 (24.3)	
Two copies	89 (2.1)	121 (2.1)	210 (2.1)	
*AD PGS*				
With APOE region‡				
pT = 0.001	0.01 (1.0)	− 0.02 (1.0)	− 0.01 (1.0)	0.51
pT = 0.01	0.00 (1.0)	− 0.02 (1.0)	− 0.01 (1.0)	0.40
pT = 0.05	− 0.01 (1.0)	− 0.02 (1.0)	− 0.02 (1.0)	0.50
pT = 0.1	− 0.01 (1.0)	− 0.02 (1.0)	− 0.02 (1.0)	0.47
pT = 0.3	− 0.02 (1.0)	− 0.02 (1.0)	− 0.02 (1.0)	0.87
pT = 1	− 0.01 (1.0)	− 0.02 (1.0)	− 0.02 (1.0)	0.71
Without APOE region^‡^				
pT = 0.001	0.01 (1.0)	− 0.02 (1.0)	− 0.01 (1.0)	0.36
pT = 0.01	0.00 (1.0)	− 0.02 (1.0)	− 0.01 (1.0)	0.42
pT = 0.05	− 0.01 (1.0)	− 0.02 (1.0)	− 0.01 (1.0)	0.52
pT = 0.1	− 0.01 (1.0)	− 0.02 (1.0)	− 0.02 (1.0)	0.48
pT = 0.3	− 0.02 (1.0)	− 0.02 (1.0)	− 0.02 (1.0)	0.88
pT = 1	− 0.01 (1.0)	− 0.02 (1.0)	− 0.02 (1.0)	0.72

	Observations			
	Male	Female	Overall	
	m = 20,802	m = 30,423	m = 51,225	P^§^
Dementia, n (%)	785 (3.8)	1078 (3.5)	1863 (3.6)	0.35
Age (yrs)	68.1 (9.8)	67.6 (10.7)	67.8 (10.3)	0.45
Stroke, n (%)	1422 (6.8)	1594 (5.2)	3016 (5.9)	0.0004
*Year, n (%)*				0.62
2002	4002 (59.9)	2682 (40.1)	6684 (13.1)	
2004	4658 (58.9)	3253 (41.1)	7911 (15.4)	
2006	4652 (59.0)	3230 (41.0)	7882 (15.4)	
2008	4531 (59.2)	3122 (40.8)	7653 (14.9)	
2010	4520 (59.1)	3128 (40.9)	7648 (14.9)	
2012	4204 (59.7)	2841 (40.3)	7045 (13.8)	
2014	3856 (60.2)	2546 (39.8)	6402 (12.5)	

SD: standard deviation; AHEAD: Asset and Health Dynamics Among the Oldest Old (b. < 1924); CODA: Children of the Depression (b. 1924–1930); HRS: Health and Retirement Study—original cohort (b. 1931–1941); WB: War babies (b. 1942–1947); EBB: early baby boomers (b. 1948–1953); MBB: mid-baby boomers (b. 1954–1959); AD: Alzheimer’s disease; PGS: Polygenic score; pT: *P-value* threshold for SNP-outcome association from the Alzheimer’s disease meta-analysis for inclusion into the polygenic score. Means and (standard deviations) are reported unless otherwise noted

^†^*P*-values are for tests of mean difference (t-test) or difference in distribution (chi-square), by sex

^§^*P*-values for dementia and stroke are from a repeated measures model with a binary distribution and logit link, unstructured correlation structure and repeated individual model to test for differences by sex. For Age, a two-step process where the mean age for each person across all visits was calculated, and then a t-test was performed on the resulting individual-means by sex

^‡^*APOE* region defined as chromosome 19 (45,384,477 to 45,432,606, build 37/hg 19). This represents the start position of *TOMM40* (45,394,477) − 10 KB and the stop position of *APOC1* (45,422,606) + 10 KB

In adjusted, repeated measures regression models, increased age, later year of observation, history of stroke, and lower education were associated with increased odds of dementia in all models (all *P* < 0.0001; Table 2). In the models using an Alzheimer’s disease PGS with the *APOE* region included, having one copy of *APOE-ε4* increased the odds of dementia relative to normal cognition by roughly twofold, while two copies of *APOE-ε4* increased the odds of dementia by over fourfold (Table 2), holding all other variables constant. The Alzheimer’s disease PGS with the *APOE* region included was marginally associated with the odds of dementia relative to normal cognition only for the pT cutoff of 0.01 (OR = 1.1 95%CI 1.0 to 1.2), while the Alzheimer’s disease PGS at all other *P*-value thresholds was not significantly associated with the odds of dementia. In the models that included the Alzheimer’s disease PGS with the *APOE* region removed, we observed similar estimates for one and two copies of *APOE-ε4* as before, and a similar effect size of ~ 8% increase in the odds of dementia for every one standard deviation increase in the Alzheimer’s disease PGSs. Both the Alzheimer’s disease PGS (without the *APOE* region) at pT = 0.001 and 0.01 were significantly associated with the odds of dementia (pT = 0.001 OR = 1.1 95%CI 1.0 to 1.2 and pT = 0.01 OR = 1.1 95%CI 1.0 to 1.2). The Alzheimer’s disease PGSs at pT = (0.05, 0.1, 0.3, and 1.0) were not associated with the odds of dementia, relative to normal cognition in this sample.

**Table 2:** Odds ratios from repeated measures generalized linear models regression analyses of dementia in the Health and Retirement Study, n = 9871, m = 51,225

	pT = 0.001			pT = 0.01			pT = 0.05			pT = 0.1			pT = 0.3			pT = 1.0		
	OR (SE)	95% CI	P	OR (SE)	95% CI	P	OR (SE)	95% CI	P	OR (SE)	95% CI	P	OR (SE)	95% CI	P	OR (SE)	95% CI	P
*Polygenic score with APOE region*^*†*^																		
Age (yrs)	1.15 (1.01)	[1.14, 1.16]	< .0001	1.15 (1.01)	[1.14, 1.16]	< .0001	1.15 (1.01)	[1.14, 1.16]	< .0001	1.15 (1.01)	[1.14, 1.16]	< .0001	1.15 (1.01)	[1.14, 1.16]	< .0001	1.15 (1.01)	[1.14, 1.16]	< .0001
Sex (male)	1.13 (1.08)	[0.96, 1.32]	0.131	1.13 (1.08)	[0.96, 1.32]	0.132	1.13 (1.08)	[0.96, 1.32]	0.143	1.12 (1.08)	[0.96, 1.32]	0.148	1.12 (1.08)	[0.96, 1.32]	0.147	1.12 (1.08)	[0.96, 1.32]	0.149
Educational (yrs)	0.76 (1.02)	[0.74, 0.79]	< .0001	0.76 (1.02)	[0.74, 0.79]	< .0001	0.76 (1.02)	[0.74, 0.79]	< .0001	0.76 (1.02)	[0.74, 0.79]	< .0001	0.76 (1.02)	[0.74, 0.79]	< .0001	0.76 (1.02)	[0.74, 0.79]	< .0001
Year	1.12 (1.01)	[1.10, 1.14]	< .0001	1.12 (1.01)	[1.10, 1.14]	< .0001	1.12 (1.01)	[1.10, 1.14]	< .0001	1.12 (1.01)	[1.10, 1.14]	< .0001	1.12 (1.01)	[1.10, 1.14]	< .0001	1.12 (1.01)	[1.10, 1.14]	< .0001
Stroke	3.36 (1.10)	[2.81, 4.01]	< .0001	3.37 (1.10)	[2.82, 4.02]	< .0001	3.36 (1.10)	[2.81, 4.02]	< .0001	3.36 (1.10)	[2.81, 4.02]	< .0001	3.37 (1.10)	[2.82, 4.03]	< .0001	3.37 (1.10)	[2.82, 4.03]	< .0001
No copies of APOE ε4	−	–	–	–	–	–	–	–	–	–	–	–	–	–	–	–	–	–
One copy of APOE ε4	2.10 (1.10)	[1.74, 2.52]	< .0001	2.12 (1.09)	[1.78, 2.53]	< .0001	2.18 (1.09)	[1.83, 2.59]	< .0001	2.18 (1.09)	[1.84, 2.60]	< .0001	2.20 (1.09)	[1.85, 2.61]	< .0001	2.20 (1.09)	[1.85, 2.62]	< .0001
Two copies of APOE ε4	4.38 (1.26)	[2.80, 6.85]	< .0001	4.48 (1.25)	[2.90, 6.94]	< .0001	4.72 (1.25)	[3.07, 7.26]	< .0001	4.77 (1.25)	[3.11, 7.34]	< .0001	4.84 (1.25)	[3.15, 7.44]	< .0001	4.86 (1.25)	[3.16, 7.48]	< .0001
AD polygenic score	1.06 (1.04)	[0.98, 1.16]	0.157	1.08 (1.04)	[1.00, 1.17]	0.064	1.07 (1.04)	[0.98, 1.16]	0.136	1.07 (1.04)	[0.98, 1.17]	0.124	1.07 (1.05)	[0.98, 1.17]	0.132	1.06 (1.05)	[0.97, 1.16]	0.166
*Polygenic score without APOE region*^*†*^																		
Age (yrs)	1.15 (1.01)	[1.14, 1.16]	< .0001	1.15 (1.01)	[1.14, 1.16]	< .0001	1.15 (1.01)	[1.14, 1.16]	< .0001	1.15 (1.01)	[1.14, 1.16]	< .0001	1.15 (1.01)	[1.14, 1.16]	< .0001	1.15 (1.01)	[1.14, 1.16]	< .0001
Sex (male)	1.13 (1.08)	[0.97, 1.32]	0.124	1.13 (1.08)	[0.97, 1.32]	0.129	1.13 (1.08)	[0.96, 1.32]	0.142	1.12 (1.08)	[0.96, 1.32]	0.148	1.12 (1.08)	[0.96, 1.32]	0.147	1.12 (1.08)	[0.96, 1.32]	0.149
Educational (yrs)	0.76 (1.02)	[0.74, 0.79]	< .0001	0.76 (1.02)	[0.74, 0.79]	< .0001	0.76 (1.02)	[0.74, 0.79]	< .0001	0.76 (1.02)	[0.74, 0.79]	< .0001	0.76 (1.02)	[0.74, 0.79]	< .0001	0.76 (1.02)	[0.74, 0.79]	< .0001
Year	1.12 (1.01)	[1.10, 1.14]	< .0001	1.12 (1.01)	[1.10, 1.14]	< .0001	1.12 (1.01)	[1.10, 1.14]	< .0001	1.12 (1.01)	[1.10, 1.14]	< .0001	1.12 (1.01)	[1.10, 1.14]	< .0001	1.12 (1.01)	[1.10, 1.14]	< .0001
Stroke	3.36 (1.09)	[2.81, 4.01]	< .0001	3.37 (1.10)	[2.82, 4.02]	< .0001	3.36 (1.10)	[2.81, 4.01]	< .0001	3.36 (1.10)	[2.81, 4.02]	< .0001	3.37 (1.10)	[2.82, 4.02]	< .0001	3.37 (1.10)	[2.82, 4.02]	< .0001
No copies of APOE ε4	–	–	–	–	–	–	–	–	–	–	–	–	–	–	–	–	–	–
One copy of APOE ε4	2.16 (1.09)	[1.82, 2.56]	< .0001	2.18 (1.09)	[1.83, 2.59]	< .0001	2.2 (1.09)	[1.85, 2.62]	< .0001	2.21 (1.09)	[1.86, 2.62]	< .0001	2.21 (1.09)	[1.86, 2.63]	< .0001	2.21 (1.09)	[1.86, 2.63]	< .0001
Two copies of APOE ε4	4.64 (1.24)	[3.03, 7.11]	< .0001	4.72 (1.24)	[3.07, 7.25]	< .0001	4.84 (1.25)	[3.15, 7.43]	< .0001	4.87 (1.25)	[3.17, 7.49]	< .0001	4.91 (1.25)	[3.19, 7.55]	< .0001	4.92 (1.25)	[3.20, 7.56]	< .0001
AD polygenic score	1.09 (1.04)	[1.00, 1.17]	0.038	1.09 (1.04)	[1.01, 1.18]	0.028	1.07 (1.04)	[0.99, 1.17]	0.095	1.08 (1.04)	[0.99, 1.17]	0.094	1.07 (1.05)	[0.98, 1.17]	0.111	1.07 (1.05)	[0.98, 1.16]	0.144

All models were additionally adjusted for five ancestry-specific principal components. Generalized linear models accounted for repeated measures by individual and used a binomial distribution, logit link, and unstructured correlation structure

OR: Odds ratio estimate for dementia relative to normal cognition; SE: standard error; CI: confidence interval; P: *P-value* for the OR estimate; *APOE*: Apolipoprotein E; AD: Alzheimer’s disease; pT: *P-value* threshold for SNP-outcome association from the Alzheimer’s disease meta-analysis for inclusion into the polygenic score

^†^*APOE* region defined as chromosome 19 (45,384,477 to 45,432,606, build 37/hg 19). This represents the start position of *TOMM40* (45,394,477) − 10 KB and the stop position of *APOC1* (45,422,606) + 10 KB

## Sensitivity analysis

Researchers have demonstrated some amount of mortality selection in the oldest individuals in the HRS genetic sample [27], and so we removed the two oldest cohorts (AHEAD and CODA) as a sensitivity analysis (Additional file 1: AF Table 2). We removed a total of 2020 individuals and 8957 observations. The effect of *APOE-ε4* remained highly associated with the odds of dementia compared to normal cognition. Though the effect size for the Alzheimer’s disease PGSs at each pT and whether or not the *APOE* region was included were not significantly different from those in Table 2, the *P*-values associated with these effects were non-significant for all Alzheimer’s disease PGS. The slightly attenuated effects are not surprising as the younger cohorts are just now entering ages at which dementia becomes more prevalent. We additionally regressed out the effect of the *APOE-ε4* from each PGS and performed the same analyses as in Table 2 (Additional file 1: AF Table 3). The PGSs with *APOE-ε4* regressed out correlated with their counterparts at a Pearson’s correlation coefficient r > 0.97 (Additional file 1: AF Table 1). As expected, the results from Table 2 and these new analyses in Additional file 1: AF Table 3 are virtually identical. Associations between each PGS and dementia without adjustment for *APOE-ε4*, and also the association between the three level *APOE-ε4* variable and dementia without adjustment for PGS are found in Additional file 1: AF Tables 4 and 5, respectively. Each of these models are adjusted for age, sex, education, year of visit, and stroke history. We found no statistical interaction between *APOE-ε4* and a PGS at a *P*-value threshold of 0.01, with [(OR_PGS*APOE(1)_ = 0.99 95%CI 0.83 to1.17; OR_PGS*APOE(2)_ = 1.05 95%CI 0.69 to 1.59] or without [(OR_PGS*APOE(1)_ = 1.00 95%CI 0.85 to 1.19]; [OR_PGS*APOE(2)_ = 1.12 95%CI 0.74 to 1.68] the *APOE* region (Additional file 1: AF Tables 6).

## Discussion

In a large, population-based cohort of older, European ancestries Americans, cumulative genetic risk summarized as a PGS is informative of longitudinal dementia odds. We observed that the *APOE* region requires handling with care in the development of PGS. Specifically, including the *APOE* region as weighted SNPs in a PGS was insufficient to account for the large risk attributed to the *APOE* region. We recommend removing the region in linkage disequilibrium around the *APOE* locus from the PGS and treating the *APOE* locus as an independent covariate. In addition, we observed greater performance from PGS developed at a *P*-value threshold of 0.01 for SNP inclusion, with greater noise from a PGS informed by the full genome in association with this dementia phenotype. Optimized measures of the polygenic nature of dementia allow for more powerful interrogations of genetic and environmental risk for dementia.

We observed the *APOE-ε4* allele was longitudinally associated with higher risk of dementia, in a dose dependent manner. This observation is consistent with extensive prior research [28, 29]. The *APOE-ε4* allele is neither necessary, nor sufficient to cause dementia, but the magnitude of increased risk attributed to each copy of the allele is relatively high. The *APOE-ε4* allele is in linkage disequilibrium with a ~ 100 kilobase region involving the *APOE*, *APOEC*, and *TOMM40* genes. Thus, an *APOE* independent PGS would need to remove the SNPs from the entire *APOE* region. In excess of the association between the *APOE* locus and dementia, we observed a small, but significant association between Alzheimer’s disease PGS and dementia. We also found that the effect of the PGS on dementia was not significantly different by *APOE* status. These findings are similar to those observed in clinical populations investigating *APOE* independent PGS risk of Alzheimer’s disease specifically [15, 30–33]. When building PGS, it is important to have independent study samples between the discovery GWAS and the application PGS. Notably, our study sample was not part of the Kunkle GWAS that generated the weights for the PGS. Our findings show the *APOE* independent Alzheimer’s disease PGS can be successfully implemented in population-based research of a broad dementia phenotype.

Dementia is a disorder with a strong genetic locus of effect (*APOE*) and substantially weaker effects are scattered throughout the genome. Including the *APOE* region in PGS without specific measurement of *APOE-ε4* is insufficient, and overestimates the polygenic nature of dementia. Similarly, in Amyotrophic Lateral Sclerosis there is a strong main effect locus (*C9orf72*). Further, there is a significant, albeit modest, proportion of the phenotypic variance explained by a polygenic risk score over and above the *C9orf72* region [34]. In contrast, other chronic disease traits, such as obesity, lack a dominant genetic locus and polygenic score development is successful across the entire genome at a higher p-value threshold [35]. Together, these results suggest that in traits with a strong genetic locus, polygenic scores should exclude the primary regions and seek to aggregate the remaining genetic risk as a separate predictor.

We acknowledge several limitations in this research. The first is that our study relied on imputed *APOE* variant calls. The *APOE* region is notoriously difficult and labor intensive to measure genotypes [36, 37]. Indeed, the two primary *APOE* SNPs of interest failed quality control metrics on the genotyping array in the HRS. We used the correlation structure of the genome from the 1000GP reference to impute these SNPs with ~ 99% confidence. Second, our study may be subject to mortality selection; however, dementia is primarily a disease of older age and requires survival long enough to manifest symptoms. Mortality selection related to the *APOE* genotype would only serve to make our observations more conservative. Third, we developed our PGS using weights from a GWAS of primarily European ancestry participants, thereby limiting generalizability to other ancestries. Last, our population-based study assessed a broad phenotype of dementia. There are many types of dementia including Alzheimer’s, vascular, and frontotemporal lobe, which have varying genetic architectures, to which we applied a PGS specific for Alzheimer’s. As future GWAS become available for dementia subtypes in a clinical population, investigators may be able to classify the utility of PGS in dementia subtypes. Future family-based studies may also consider SNPs strongly associated with early-onset AD, including *APP*, *PSEN1*, *or PSEN2* mutations.

## Conclusion

Dementia has considerable risk attributed to genetic factors. The *APOE* region is the strongest locus associated with disease and many additional sites confer small risk effects. Incorporating genetic risk from many sites in a polygenic risk score is a useful metric for risk prediction and etiologic testing in epidemiologic research of complex traits [38]. Our findings demonstrate the *APOE* region should be removed prior to polygenic risk score development and treated as an independent factor in dementia analyses. More work is needed to assess polygenic scores for Alzheimer’s disease for clinical utility and prediction and in diverse ancestries.

## Supplementary information

**Additional file 1.** Supplemental tables.

## Data Availability

Phenotype and covariate data are publicly available through the Health and Retirement Study, public use dataset. Produced and distributed by the University of Michigan with funding from the National Institute on Aging (Grant Number NIA U01AG009740) [39]. https://hrs.isr.umich.edu/data-products. The data/analyses presented in the current publication have been deposited in and are available from the dbGaP database under dbGaP accession phs000428.v2.p2 [40]. Summary statistics for Kunkle et al. [6] are available through The National Institute on Aging Genetics of Alzheimer's Disease Data Storage Site (https://www.niagads.org/datasets/ng00075). The list of specific genetic variants included in each polygenic score during the current study are available from the corresponding author on reasonable request.

## Abbreviations

AD: Alzheimer’s disease
APOE: Apolipoprotein E
CI: Confidence interval
GWAS: Genome-wide association study
HRS: Health and Retirement Study
IGAP: International Genomics of Alzheimer’s Project
OR: Odds ratio
PGS: Polygenic risk score
PC: Principal component
pT: P-value threshold
SNP: Single nucleotide polymorphism
1000GP: 1000 Genomes Project
